# Functional outcome after treatment of TB of upper limbs

**DOI:** 10.1186/1753-6561-9-S3-A100

**Published:** 2015-05-19

**Authors:** Ida S Tacata

**Affiliations:** 1 University of the Philippines, Manila, Philippines

## 

Musculoskeletal tuberculosis manifest commonly in the spine, hip and knee and rarely in the hand and wrist. Certain factors influence the functional outcome after treatment of tuberculosis of the upper extremity namely location (proximal or distal), tissue involvement, delay prior to consult, care by a multispecialty team and early surgery.

Appropriate drug therapy is the mainstay in the management of musculoskeletal tuberculosis. The role of medical management of tuberculosis of the upper extremity appear to have a predominant role compared to surgery with the latter reserved for drainage of abscesses, synovectomy and carpal tunnel release.

The functional outcome with medical treatment alone in the series of Kotwal and Khan (2010) was good using the modified Green and O'Brien scoring system. Benkeddache (1982) reported favorable response to drug treatment with resolution of bone and soft tissue lesions. There is a scarcity of reports in this area prompting the author to undertake this study in 2013-2014 on 25 Filipino subjects.

The functional outcome of patients with tuberculosis of the upper extremity aged 17 to 87 years who received appropriate medical and surgical treatment for tuberculosis of the upper extremity was determined using the DASH scoring system (Filipino version) . The majority of the patient population had surgical treatment which included wrist fusion, finger joint fusion, joint synovectomy, tendon synovectomy, arthrotomy, arthroscopic debridement and ray amputation.

The mean DASH score of patients with tuberculosis of the upper extremity was 28. This score allowed patients to do ADLs without much difficulty. The type of tissue involvement and the location involved did not appear to be good predictors of functional outcome after medical and surgical treatment of tuberculosis of the upper extremity.

